# Comparative analysis of the cytotoxic effect of 7-prenyloxycoumarin compounds and herniarin on MCF-7 cell line

**Published:** 2015

**Authors:** Seyed Hadi Mousavi, Atiyeh-Sadat Davari, Mehrdad Iranshahi, Sarvenaz Sabouri-Rad, Zahra Tayarani Najaran

**Affiliations:** 1 *Medical Toxicology Research Centre, Mashhad, University of Medical Sciences, Mashhad, Iran. *; 2 *Pharmacological Research Centre of Medicinal Plants, School of Medicine, Mashhad, University of Medical Sciences, Mashhad, Iran*; 3 *Biotechnology Research Center, School of Pharmacy, Mashhad University of Medical Sciences, Mashhad, Iran*; 4 *Department of Pharmacodynamics and Toxicology, School of Pharmacy; Mashhad University of Medical Sciences, Mashhad, Iran*

**Keywords:** *Apoptosis*, *Bax*, *Cytotoxicity*, *MCF-7*, *7-Prenyloxycoumarins*

## Abstract

**Objective:**

7-prenyloxycoumarins are a group of secondary metabolites that are found mainly in plants belonging to the Rutaceae and Umbelliferae families. This study was designed to evaluate and compare the cytotoxic and apoptotic activity of 7-prenyloxycoumarin compounds and herniarin on MCF-7, a breast carcinoma cell line.

**Materials and Methods:**

Cells were cultured in RPMI medium and incubated with different concentrations of auraptene, herniarin, umbelliferone, and umbelliprenin. Cell viability was quantified by MTT assay. Apoptotic cells were determined using propidium iodide staining of DNA fragmentation by flow cytometry (sub-G1peak). Bax protein expression was detected by western blot to investigate the underlying mechanism.

**Results:**

Doses which induced 50% cell growth inhibition (IC_50_) against MCF-7 cells with auraptene, herniarin, umbelliferone, and umbelliprenin were calculated (59.7, 207.6, 476.3, and 73.4 µM), respectively. Auraptene induced a sub-G1 peak in the flow cytometry histogram of treated cells compared to control cells, and DNA fragmentation suggested the induction of apoptosis. Western blot analysis showed that auraptene significantly up-regulated Bax expression in MCF-7 cells compared to untreated controls.

**Conclusion:**

Auraptene exerts cytotoxic and apoptotic effects in breast carcinoma cell line and can be considered for further mechanistic evaluations in human cancer cells. These results candidate auraptene for further studies to evaluate its biosafety and anti-cancer effects.

## Introduction

Apoptosis is a sequence of regulated molecular events induced by many of the chemotherapeutic agents used in cancer treatment (Poncet and Kroemer, 2002 ▶; Mousavi et al., 2008 ▶). The induction of apoptosis in tumor cells is considered very useful in management and therapy as well as in the prevention of cancer. A wide variety of natural substances has been documented to have the ability to induce apoptosis in different tumor cells. In this regard, screening apoptotic inducers of plants, either in the form of crude extracts or as components isolated from them, is very important (Cassileth et al., 2008 ▶).

A natural product with interesting pharmacological properties is coumarin (benzopyran-2-one or chromen-2-one) ring. Many interesting coumarin derivatives have been synthesized including the furanocoumarins, pyranocoumarins, and coumarin sulfamates (COUMATES), which have been found to be useful in photo-chemotherapy, antitumor, and anti-HIV therapy, as a stimulant for the central nervous system, and as antibacterial, anti-inflammatory, and anti-coagulant drugs (Musa et al., 2008 ▶). 

**Figure 1:**
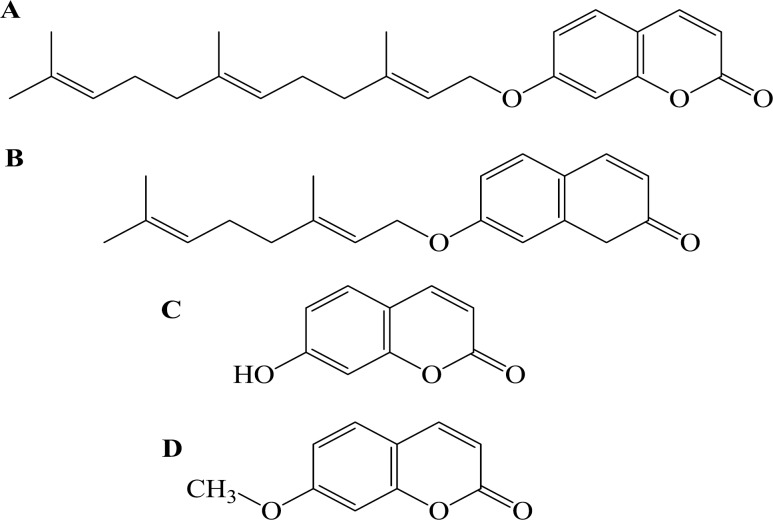
Chemical structures of 7-prenyloxycoumarins: umbelliprenin (A), auraptene (B) umbelliferone (C), and herniarin (D).

Various biological and pharmacological activities of 7-prenyloxycoumarins, a group of secondary metabolites found in families of Rutaceae and Umbelliferae, have gained the attention of researchers in the last two decades (Askari et al., 2009 ▶). Some of the most studied prenylated coumarins include auraptene, herniarin, umbelliferone, and umbelliprenin. Auraptene (7-geranyloxycoumarin) (Figure 1B) is the most abundant geranyloxycoumarin obtained from some species of the genus *Citrus* (Curini et al., 2004 ▶) with known cancer chemopreventive (Tanaka et al., 1998 ▶) and anti-tumor properties against many types of cancers (Sakata et al., 2004 ▶; Murakami et al., 2000 ▶). In addition, it possess anti-inflammatory (Curini et al., 2004 ▶; Murakami et al., 2000 ▶) and spasmolytic activities (Yamada et al., 1997 ▶) and can suppress the release of tumor necrosis factor alpha (TNF-α) (Tanaka et al., 1999 ▶), superoxide anion generation by inflammatory leukocytes (Murakami et al., 2000 ▶) and IкB (nuclear factor of kappa light polypeptide gene enhancer in B-cells inhibitor) degradation (Murakami et al., 2003 ▶). Auraptene also has the ability to inhibit the platelet aggregation induced by arachidonic acid and platelet activated factor *in vitro* (Chen et al., 1995 ▶). 

Herniarin (Figure 1D) is a methoxy analogue of umbeliferone occuring naturally in some flowering plants (Ma et al., 2007 ▶). Herniarin was reported to have anti-dermatophytic activity (Mares et al., 1993 ▶). 

Umbelliprenin is a prenylated coumarin (Figure 1A) and is found in various plant species such as Ferula (Umbellifera) species, *Angelica archangelica *(Linn.)*, Coriandrum sativum *L., and* Citrus limon *(L.) Osbeck, which are consumed as food or used for food preparation. Previous researches have shown various pharmacological activities including inhibition of red pigment production in *Serratia marcescens* (Iranshahi et al., 2004 ▶), inhibition of squalene-hopene cyclase (SHC) (an enzyme taking part in sterol synthesis) (8), decreasing matrix metaloproteinase (MMP) activity (Baba et al., 2002 ▶), anti-leishmanial activity against promastigotes (Iranshahi et al., 2007 ▶), apoptosis induction in human M4Beu metastatic pigmented melanoma cells (Barthomeuf et al., 2008 ▶), and cancer chemopreventive activity (Iranshahi et al., 2008 ▶).

Umbelliferone (Figure 1C) is a synthesized chemical with the potential to be antimutagenic/ anticarcinogenic against mutations induced by benzo (a) pyrene, a potent mutagen/carcinogen, and hydrogen peroxide. It has also the ability to function as free radical scavengers. 

Some coumarins and their active metabolite, 7-hydroxycoumarin analogs, have also shown inhibitory activity on breast cancer tumor cells (Musa et al., 2008 ▶). 

Herein, we investigated the cytotoxic and proapoptotic effects of synthesized 7-prenyloxycoumarins and herniarin as bioactive natural coumarins in MCF-7 cells as a widely-used model system for the study of breast cancer (Simstein et al., 2003 ▶). We also explored the role of Bax protein in aurapten-induced apoptosis in MCF-7 cells.

## Material and Methods

**Preparation of 7-prenyloxycoumarins compounds and herniarin**

Auraptene, herniarin, umbelliferone, and umbelliprenin were synthesized according to the previously described method (Askari et al., 2009 ▶). Bioactive 7-prenyloxycoumarins, namely, auraptene, umbelliprenin, and together with herniarin were synthesized from 7-hydroxycoumarin under alkaline conditions and then purified by column chromatography. The structures of the products were characterized by NMR spectroscopic method including 1H- and 13C-NMR experiments. For antiproliferative assay, auraptene, herniarin, umbelliferone, and umbelliprenin were diluted in DMSO. Immediately before use, they were diluted in the culture medium to obtain a final DMSO concentration of 0.5% (v/v).

**Chemicals and reagents**

RPMI-1640 medium and fetal bovine serum were purchased from Gibco (London, UK); 3-(4, 5-dimethylthiazol-2-yl)-2 and 5-diphenyl tetrazolium (MTT) from Promega (Madison, WI, USA); ethidium bromide, RNase A, and Proteinase K from Fermentas (Ontario, Canada); Bax antibody, β-actin antibody, FAS-L antibody, anti-rabbit IgG, and HRP linked antibody from CellSignaling technology (Boston, USA); ECL Western blotting detection reagent from Bio-RaD (USA).

**Cell cultures and treatment agent**

The human breast cancer cells (MCF-7) were maintained in RPMI-1640 medium supplemented with 10% fetal bovine serum at 37 ºC in a humidified atmosphere of 95% air and 5% CO_2_. The stock solution of compounds was prepared at 100 mg/ml in dimethylsulfoxide and kept at –20 °C. For MTT assay, cells were seeded at 10^4^ cells per well onto 96-well culture plates. For assay of apoptosis, cells were seeded at 10^5^ cell per well onto a 24-well plate.

For each concentration and time course study, there was a control sample that remained untreated which received the equal volume of medium.

**Cell viability**

Cell viability was determined using a modified MTT assay (Mosmann et al., 1983 ▶; Mousavi et al., 2010 ▶). Briefly, the cells were seeded (10^4^ cells per well) onto flat-bottomed 96-well culture plates and allowed to grow 72 h after treatment with various concentration of auraptene, herniarin, umbelliferone, and umbelliprenin. After removing the medium, MTT solution (5 mg/ml in PBS) was added and incubated for 4 h and the resulting formazan was solubilized with DMSO (100 ml). The absorption was measured at 570 nm (620 nm as a reference) in an ELISA reader. 

**Cell morphology**

The MCF-7 cells were plated in 96-well plates at a density of 10^4^ cells/well and grown for 48 h in order to attach to the surface of the plates completely. Auraptene, herniarin, umbelliferone, and umbelliprenin were added in different concentrations (0, 25, 50, and 100 µM) to the cells and then the cells were grown at 37 °C in a humidified atmosphere with 5% CO_2_ for 48 h. For cell morphology experiments, the culture plates were examined and photographed by the inverted light microscope.

**PI Staining**

Apoptotic cells were detected using PI staining of treated cells followed by flow cytometry to detect the so-called sub-G1 peak (Tayarani-Najaran et al., 2010 ▶). It has been reported that DNA fragmentation creates small fragments of DNA that can be eluted following incubation in a hypotonic phosphate-citrate buffer. When stained with a quantitative DNA-binding dye such as PI, cells that have lost DNA will take up less stain and will appear to the left of the G1 peak. Briefly, 10^5^ MCF-7 cells were seeded in each well of a 24-well plate and treated with IC_50_ concentrations: 70, 80, and 200 µM of auraptene, umbelliprenin, and herniarin, respectively for 48 h. MCF-7 cells were also treated with various concentrations of auraptene for 48 h. Floating and adherent cells were then harvested and incubated at 4 °C overnight in the dark with 750 μL of a hypotonic buffer (50 μg/mL PI in 0.1% sodium citrate plus 0.1% Triton X-100) before flow cytometric analysis using a FACScan flow cytometer (Becton Dickinson). 10^4^ events were acquired with FACS. 

**DNA fragmentation**

Isolation of apoptotic DNA fragments was performed based on the modified method reported by Gong et al. (Akins et al., 1992 ▶). In brief, the MCF-7 cells were incubated with 70, 80, and 200 µM of auraptene, umbelliprenin, and herniarin, respectively for 48 h. At the end of incubation, cells were centrifuged, washed twice with ice-cold PBS, and a volume equal to 4×10^6^ cells of either type fixed overnight in 70° ethanol in -20 °C. The cells were centrifuged at 800 g for 5 min and the ethanol was removed. 

The cell pellet was resuspended in 40 µl of phosphate-citrate buffer (PCB) at room temperature for 30 min. After centrifugation at 1000 g for 5 min, the supernatant was then incubated with 4 µl of 0.25% Nonidet P-40 followed by 3 µl of 1 mg/ml RNase A at 37 °C for 30 min. Three µl of 1 mg/ml proteinase K was added and the extract was incubated for an additional 30 min at 37 °C. An equal amount of this sample (20 ml) was electrophoresed through 1% agarose gel containing ethidium bromide in TBE buffer (45 mM Tris-borate, 1 mM EDTA) at 60 V for 2 h.

**Western blotting analysis**

MCF-7 cells were treated with 100 µM of auraptene, umbelliprenin, and herniarin for 48 h. The cells were harvested and rinsed with ice-cold PBS. The cell pellet was resuspended in a lysis buffer containing 50 mM Tris-HCl (pH 7.4), 150 mM NaCl, 1% triton-X 100, 1 mM EDTA, 0.2% SDS, 1% protease inhibitor cocktail, 1% phosphatase inhibitor cocktail, and 1 mM phenylmethylsulfonyl fluoride, and left on ice for 30 min. After centrifugation at 10000 rpm for 20 min at 4 °C, the cell lysate was collected and protein concentration was determined according to the BCA detection kit (Gong et al., 1994). Equal amounts of proteins were subjected to 12.5% SDS–PAGE (w/v). The proteins were transferred to PVDF membrane and subjected to immune-blotting using Bax antibody and β-actin antibody, as primary antibodies, and anti-rabbit HRP linked IgG as secondary antibodies. Bax protein bands in MCF-7 cells were detected by enhanced chemiluminescence using the ECL Western blotting detection reagent. Scanned autorad images were quantified using Quantpro software (Molecular Dynamics).

**Statistical analysis**

One way analysis of variance (ANOVA) and Bonferroni’s post hoc were used for data analysis. All results were expressed as mean ± SEM and *p-*values below 0.05 were considered statistically significant. 

## Results

**Inhibition of cell viability**

Inhibition of cell viability caused by prenylated coumarins including auraptene, herniarin, umbelliferone, and umbelliprenin was examined using MTT assay. 

**Figure 2:**
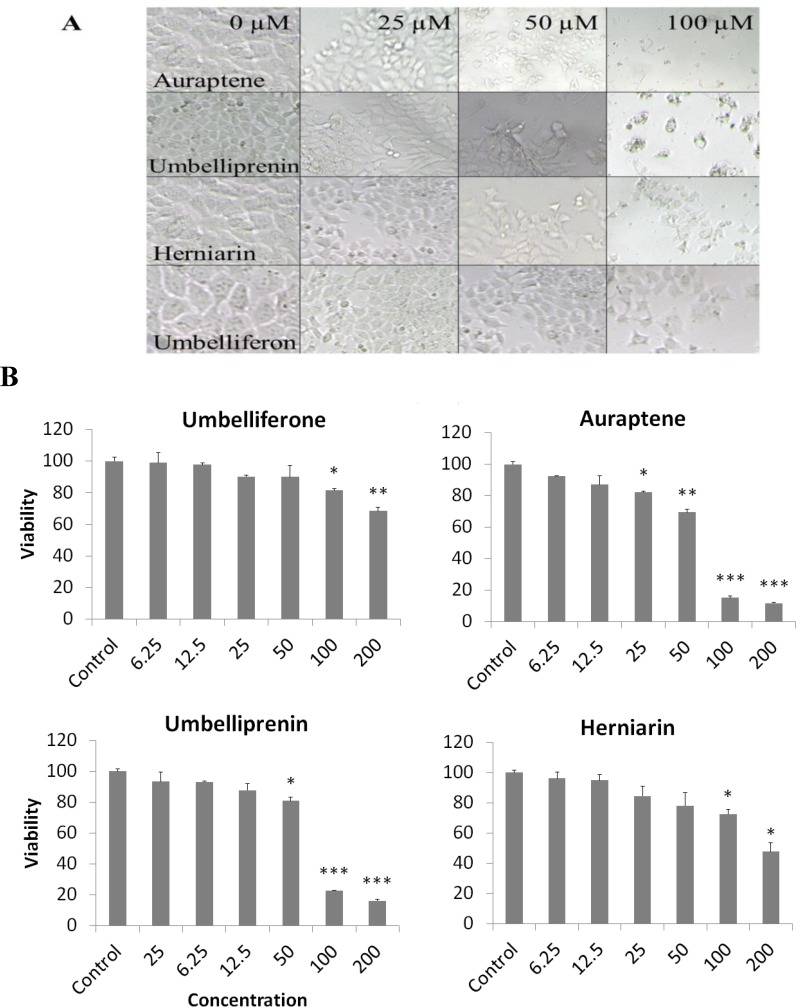
(A). Morphological changes of MCF-7 cells after treatment with auraptene, herniarin, umbelliferone, and umbelliprenin for 48 h. Control cells remained untreated and received an equal volume of the solvent. (B). Cytotoxic effects of auraptene, herniarin, umbelliferone, and umbelliprenin on MCF-7 cells. Cells were treated for 72 h with different concentrations of prenylated coumarins. Cytotoxicity was determined by MTT assay. Results are the mean ± SEM of three independent experiments. ∗p <0.05, ∗∗p <0.01, and ∗∗∗p <0.001 compared to control

In order to compare the cytotoxicity of auraptene, herniarin, umbelliferone, and umbelliprenin, the MCF-7 cells were incubated with different concentrations of prenylated coumarins for 72 h. The results showed that these compounds decreased cell viability of cells in a concentration-dependent manner. The doses inducing 50% cell growth inhibition (IC_50_) against MCF-7 cells for auraptene, herniarin, umbelliferone, and umbelliprenin were calculated to be 59.7, 207.6, 476.3,and 73.4 µM, respectively (Figure 2). This toxicity was associated with morphological changes including reduction of cell volume and rounding of the cells. The substantial morphological changes observed in auraptene-treated MCF-7 cells were examined and photographed by the inverted light microscope. Damaged cells became round and shrunken, while the untreated cells retained the normal size and shape (Figure 2).

**Apoptosis induction by auraptene, umbelliprenin, and herniarin in MCF-7 cells**

**Figure 3:**
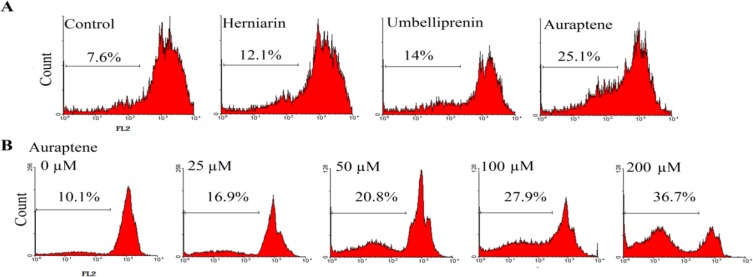
(A). Flow cytometry histograms of apoptosis assays by PI method in MCF-7 cells. Cells were treated with 70, 80, and 200 µM, auraptene, umbelliprenin, and herniarin, respectively for 48 h. Sub-G1 peak as an indicative of apoptotic cells, was induced in prenylated coumarins-treated but not in control cells. (B). Flow cytometry histograms of apoptosis assays by PI method in MCF-7 cells. Cells were treated with different concentrations of auraptene for 48 h. Sub-G1 peak as an indicative of apoptotic cells, was induced in auraptene-treated but not in control cells

Apoptosis following treatment with auraptene, umbelliprenin, and herniarin was measured with PI staining and flow cytometry aiming to detect the sub-G1 peak resulting from DNA fragmentation. The MCF-7 cells treated with 100 µM auraptene, umbelliprenin, and herniarin for 48 h induced a sub-G1 peak in flow cytometry histogram compared to untreated control cells (Figure 3A).

According to the results, we treated the cell line with different concentrations of auraptene for 48 h. The results indicated the involvement of an apoptotic process in auraptene-induced cell death in a concentration-dependent manner (Figure 3B).

**Figure 4:**
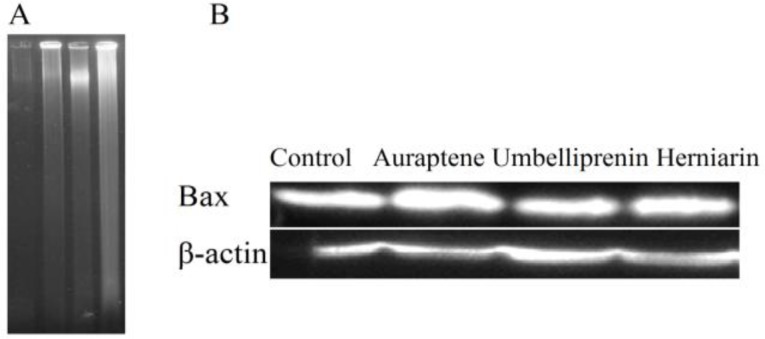
A). Internucleosomal fragmentation of cells treated with auraptene, umbelliprenin, and herniarin for 48 h. After harvesting the cells, isolated DNA was analyzed by agarose gel elctrophoresis. Lane 1, untreated cells; lanes 2, 3, and 4 cells treated with 70, 80, and 200 µM, auraptene, umbelliprenin, and herniarin, respectively. (B). Western blot analysis of Bax protein expression in MCF-7 cells. Lane 1, untreated cells; lanes 2, 3, and 4 cells treated with 70, 80, and 200 µM, auraptene, umbelliprenin, and herniarin, respectively for 48 h. β-actin was used as a loading control

DNA fragmentation was also observed in MCF-7 cells, which confirmed apoptosis induction in this cell line (Figure 4A).

**Expression of Bax proteins**

Bax proteins play a pivotal role in controlling cytochrome c release and apoptosis initiation via the mitochondrial pathway (Gross et al., 1999 ▶). Auraptene in IC_50_ (60 µM) enhanced the expression of Bax protein in MCF-7 cells (Figure 4B).

## Discussion

Breast cancer is the second leading cause of cancer-related death in women (Patil et al., 2010 ▶). The current treatment modalities of breast cancer causes leukemia, nausea, vomiting, blood clots endometrial cancer, and cardiotoxicity as side effects. 

Hence, chemoprevention of cancer could be a better strategy. Pharmacological properties of bioactive compounds in cancer treatments and prevention have developed many natural-based cancer therapeutics (Scott et al., 2009 ▶). 

7-prenyloxycoumarins are a group of compounds which have been shown to possess preventive and therapeutic effects on breast cancer but they have also cytotoxic effects on some other cancer cell lines.

We have demonstrated that treatment of MCF-7 cells with auraptene, herniarin, and umbelliprenin results in a significant decrease in cell viability by inducing apoptosis. In our research, assays for cell viability demonstrated that 59.74, 73.4, 476.3, and 207.6 μM of auraptene, umbelliprenin, umbelliferone, and herniarin were the IC_50_ values against MCF-7 cancer cells at 48 h, respectively. 

Morphological characteristics of control cells, seemed to be normal and with increased concentration of these compounds, volume and number of the cells significantly decreased, chiefly with auraptene. The lowest IC_50_ and the most reduced volume in treated MCF-7 cells were related to auraptene, so it is the most potent inhibitor of MCF-7 growth compared to the other compounds. The cytotoxicity of auraptene started in concentration of 25 μM and at the concentration of 100 μM it could decrease the viability to below 20% (Figure 2B). 

Literature has shown the cytotoxicity and chemo-preventive properties of umbelliprenin (Iranshahi et al., 2008 ▶; Iranshahi et al., 2009 ▶). Previous studies with umbelliprenin towards two human colon cancer cell lines, HT-29 and HCT 116, and breast cancer cells showed weak cytotoxic activities (Barthomeuf et al., 2008 ▶; Jabrane et al., 2010 ▶; Khorramizadeh et al., 2010 ▶). 

It has been demonstrated that diet supplementation with auraptene *in vivo* has the potency to reduce the growth and number of metastatic lung tumors (Tanaka et al., 2000 ▶). Recently, an *in vivo* study also showed that dietary auraptene (500 ppm in the diet) decreased mammary carcinoma incidence and delayed median time to tumor in N-methylnitrosourea (MNU) treated rats (Krishnan et al., 2009 ▶). In two different studies, de Medina et al., and Krishnan et al., reported that auraptene induces concentration-dependent growth control through an arrest of the cell cycle in the G0-G1 phase in MCF-7, MDAMB-231, SW-620, and E151A cell lines, showing that auraptene inhibits both the proliferation of ER+ (MCF-7) and ER- (MDA-MB-231) human breast cancer cells (de Medina et al., 2010 ▶; and Krishnan et al., 2009 ▶). In addition, auraptene reduces cell survival in a clonogenic assay and also inhibits invasiveness. These data establish that auraptene reduces the proliferation, viability, and invasiveness of tumor cells of different origins (de Medina et al., 2010 ▶). 

Barthomeuf et al., previously reported the proliferation of M4Beu cells is more potently inhibited by umbelliprenin (IC_50_ =12.3 μM) than by the citrus coumarin auraptene (7- geranyloxycoumarin, IC_50_ =17.1 μM) and they were capable of inhibiting the prevalence of lung metastasis in mice bearing B16BL6 murine melanoma (Barthomeuf et al., 2008 ▶). One study demonstrated that umbelliprenin has antitumor activity on both A549 and QU-DB cell lines with IC_50 _values of 59 and 47 μM for A549 and QU-DB cells, respectively (khaghanzadeh et al., 2012 ▶). Based on scientific reports, both auraptene and umbelliprenin have more toxicity on M4Beu cells in comparison with MCF7 cells and auraptene is more toxic on MCF7 cells in comparison with umbelliprenin.

Our results showed that the cytotoxicity of 7-prenyloxycoumarins is related to induction of apoptosis. Sub-G1 peak was observed in the flow cytometry histogram of breast cancer cells at the concentration of 70, 80, and 200 μM of auraptene, umbelliprenin, and herniarin, respectively at 48 h (Figure 4). The data from flow cytometry histogram of auraptene indicated the increase of sub-G1 peak in a dose-dependent manner. Induction of apoptosis in cancer cells by compounds is a key target for chemotherapeutic and chemopreventive applications. Mitochondria plays a pivotal role in apoptosis. The balance of pro-apoptotic proteins such as Bid, Bax, and Bak, and anti-apoptotic proteins including Bcl-2 and Bcl-xL, which are controlled by levels of cellular damage or stress distinguish the cell fate of apoptosis (Michael et al., 2000 ▶). Bax is a pore-forming pro-apoptotic protein that facilitates cytochrome c release and triggers caspase-mediated apoptotic cell death (Gross et al., 1999 ▶). Deficiency of Bax and Bak confers resistance to most conventional cancer therapies (Xu et al., 2015 ▶). Cells treated with auraptene increased levels of Bax, which is a key marker of mitochondrion involvement in auraptene-induced apoptosis. 

In one study, umbelliprenin exhibited cytostatic effects and reduced the serum-induced proliferation of M4Beu cells through cell cycle blockade in G1 and induction of dose-dependent apoptosis with little cytotoxicity on primary fibroblasts (Shahverdi et al., 2006 ▶). khaghanzadeh et al., demonstrated that the pattern of cell death in both A549 and QU-DB cell lines was apoptosis (khaghanzadeh et al., 2012 ▶). Some researches showed other mechanisms for 7-prenyloxycoumarins. The proved toxicity of umbelliprenin for melanoma cancer cells and its ability to decrease MMP activity and expression in cancer cells (Shahverdi et al., 2006 ▶) support the hypothesis that foods or remedies containing umbelliprenin as well as coumarin might provide protection against the development and recurrence of malignant melanoma in humans with few side effects (Barthomeuf et al., 2008 ▶). 

Auraptene reduces cyclin D1 protein which is overexpressed in human breast cancer (Krishnan et al., 2012 ▶). The effect of auraptene on cell cycle could persist for at least 24 h which could be the effect of parent compound or its active metabolites. The major metabolites of auraptene are umbelliferone and 7-ethoxycoumarin which also have been shown to possess chemopreventive effects (Murakami et al., 2000 ▶). Auraptene and umbelliprenin both have the same parent structure. The presence of an acyclic sesquiterpenene group in place of the geranyl group at C7-OH of the 1, 2- benzopyrone ring in umbelliprenin has been found to be related to the cytotoxicity of umbelliprenin (Barthomeuf et al., 2008 ▶). We conclude that the presence of a hydrophobic chain in place of the geranyl group at C7-OH plays an important role in cytotoxicity of auraptene via increasing lipophilicity and as a result, easier penetration to the cells. The absence of this group in umbelliferone, metabolite of auraptene, has caused a reduction in cell toxicity and our data confirmed this hypothesis. In comparison, herniarin with more hydrophobic group, is more cytotoxic than umbelliferone for cells. Li et al. reported the same result and the HIF-1 inhibitory activity of geranyloxycoumarins derivatives appears to be dependent upon the substitution pattern of both the coumarin backbone and the C-7 substituted geranyl side chain. Addition of certain fatty acyl chains to the basic system can increase the HIF-1 inhibitory activity. In acylated geranyloxycoumarins, membrane penetration is facilitated because of the lipophilic side chains. Auraptene, natural geranyloxycoumarin with the most lipophilic side chain, was among the most potent non-acylated HIF-1 inhibitors (Li et al., 2013 ▶).

Our results suggest that auraptene may be a potential chemotherapeutic or chemo-preventive agent based on its ability to induce apoptosis in cancer cells. While our investigation with MCF-7 cells as an *in vitro* model proposes strong evidence for auraptene-induced apoptosis, further studies with clinical trials need to be conducted to establish auraptene as a safe agent for cancer therapy.
